# Varicose Tumour of the Labia during Labour

**Published:** 1848-01

**Authors:** 


*Varicose Tumour of the Labia during Labour.*—Dr. Burns had seen several cases of enlarged varicose veins of the labia. In one instance they formed an immense tumour which burst shortly before the head passed, and caused a considerable loss of blood.

Dr. Simpson had met with the same several times. Some of the tumours were large; one only ruptured. The hæmorrhage being venous, is like venous hæmorrhage elsewhere, generally easily restrained by a little compression. If it is troublesome, the labour may be hastened by the forceps or otherwise. Dr. S. stated in proof of the very vascular character of the parts at the root of the labia externa, that three instances had occurred in Edinburgh during the last twenty years of death from cuts or stabs in the pudendal venous plexus. In all these three cases the persons inflicting the wound had been tried in our criminal courts for murder.—*Dub. Med. Press.*

